# Substituent Effects in Bioorthogonal Diels–Alder Reactions of 1,2,4,5‐Tetrazines

**DOI:** 10.1002/chem.202300345

**Published:** 2023-04-13

**Authors:** Nicole Houszka, Hannes Mikula, Dennis Svatunek

**Affiliations:** ^1^ Institute of Applied Synthetic Chemistry TU Wien Getreidemarkt 9 1060 Vienna Austria

**Keywords:** bioorthogonality, click chemistry, cycloaddition, Diels–Alder, tetrazines

## Abstract

1,2,4,5‐Tetrazines are increasingly used as reactants in bioorthogonal chemistry due to their high reactivity in Diels–Alder reactions with various dienophiles. Substituents in the 3‐ and 6‐positions of the tetrazine scaffold are known to have a significant impact on the rate of cycloadditions; this is commonly explained on the basis of frontier molecular orbital theory. In contrast, we show that reactivity differences between commonly used classes of tetrazines are not controlled by frontier molecular orbital interactions. In particular, we demonstrate that mono‐substituted tetrazines show high reactivity due to decreased Pauli repulsion, which leads to a more asynchronous approach associated with reduced distortion energy. This follows the recent Vermeeren–Hamlin–Bickelhaupt model of reactivity increase in asymmetric Diels–Alder reactions. In addition, we reveal that ethylene is not a good model compound for other alkenes in Diels–Alder reactions.

## Graphical abstract

**Non‐frontier molecular orbital interaction‐controlled reactivity**: The reactivity of 1,2,4,5‐tetrazines in inverse electron demand Diels–Alder cycloadditions is often described by using frontier molecular orbital (FMO) theory. We show that FMO interactions play a minuscule role in the reactivity difference between commonly used scaffolds. Instead, Pauli repulsion is often the deciding factor.

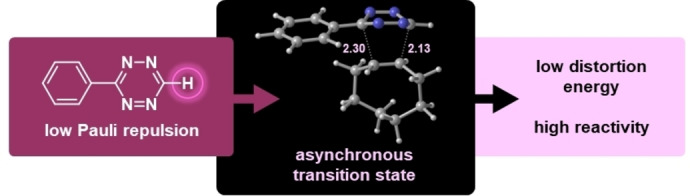

1,2,4,5‐Tetrazines react rapidly with strained alkenes, such as *trans*‐cyclooctenes, in an inverse electron‐demand Diels‐Alder cycloaddition followed by a retro‐Diels–Alder reaction with loss of nitrogen (Figure 1a).^[1]^ The resulting dihydropyridazines can tautomerize and eventually oxidize to pyridazine products. This biocompatible ligation reaction has received a lot of attention due to the high second order rate constants that can be achieved.^[2]^ Substituents in the 3‐ or 6‐position have a substantial effect on the Diels–Alder reactivity of this azine resulting in reactivity differences of several orders of magnitude.^[3]^ Commonly used scaffolds include alkyl and aryl substituents. Additionally, highly reactive mono‐substituted tetrazines are frequently employed bioorthogonal tools.^[4]^ The influence of the substituents on the tetrazine reactivity is often explained by frontier molecular orbital (FMO) theory.^[5]^ Because the rate‐limiting first step of this reaction is an inverse electron‐demand Diels–Alder cycloaddition, electron‐poor tetrazines are generally more reactive. However, there are examples where frontier molecular orbital theory cannot explain the relative reactivities of tetrazines. We recently demonstrated that the high reactivity of pyridyl‐substituted tetrazines results to a large degree from a lowered distortion energy rather than a stronger FMO interaction.^[6]^ In addition, mono‐aryl‐substituted tetrazines react faster than the corresponding di‐aryl‐substituted compounds, despite having a higher unoccupied FMO.^[7]^ Using computational methods, we demonstrate how tetrazine substituents influence the reactivity in these Diels–Alder reactions and shine light on the underlying mechanisms.

**Figure 1:**
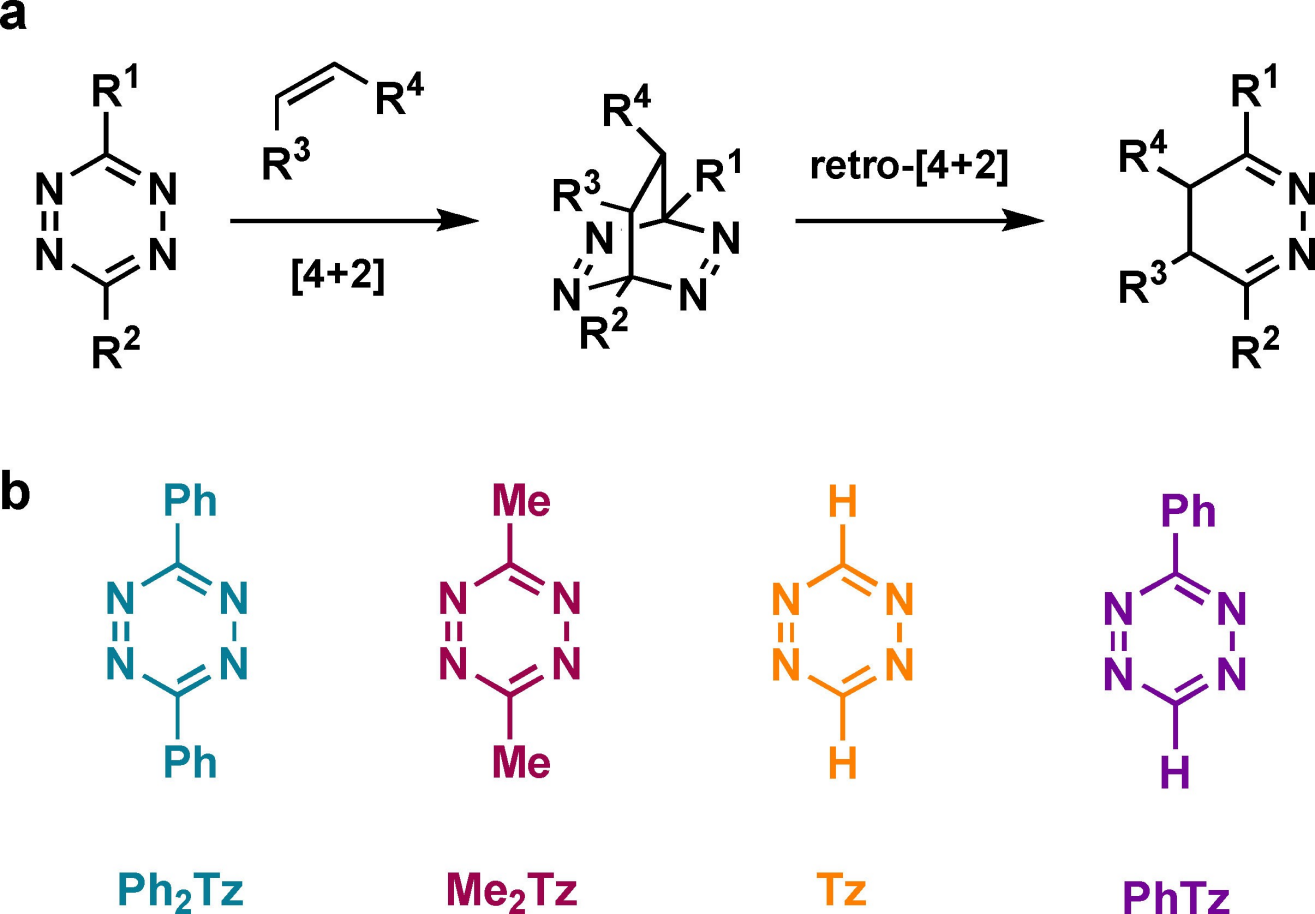
a) Mechanism of bioorthogonal tetrazine–alkene cycloadditions. b) Tetrazines used in this study.

## Computational Methods

### Computational details

Calculations were performed at the M06‐2X/def2‐TZVP level of theory using Gaussian 16 Rev A.03.^[8]^ M06‐2X is known to perform well for cycloadditions and in particular tetrazine Diels–Alder reactions.[^4b^, ^9^] A quasi classical correction was applied to entropy calculations by setting all frequencies below 100 to 100 cm^−1^ using GoodVibes.^[10]^ Energy decomposition analysis was performed in ADF (2022.101)^[11]^ using M06‐2X/TZ2P, a numerical quality of VeryGood and no frozen core. Distortion/interaction analysis was performed in ADF as part of the energy decomposition analysis and matched the results obtained in Gaussian calculated by using autoDIAS.^[12]^ EDA was performed using PyFrag 2019.^[13]^ Consistent geometry structures were achieved by performing an optimization with frozen C−C forming bond lengths using the opt=modredundant keyword in Gaussian 16. CPK coloring is used in the visualization of 3D structures.

### Distortion/interaction and energy decomposition analysis

To gain detailed and quantitative insight, we applied the distortion/interaction model in combination with energy decomposition analysis (EDA);^[14]^ methods that have been successfully used to investigate bioorthogonal reactions.[^6^, ^15^] In the distortion/interaction analysis the electronic energy Δ*E* of two interacting molecules is decomposed into the distortion energy Δ*E*_dist_ associated with distorting the reactants from their equilibrium geometry and the interaction energy Δ*E*_int_ between the distorted reactants. Δ*E*_int_ can be further analyzed using EDA to obtain three physically meaningful terms within the framework provided by the Kohn‐Sham molecular orbital theory:^[14b]^ i) Δ*V*_elstat_ describes the electrostatic interaction between the unperturbed charge distributions of the distorted molecules; ii) Δ*E*_oi_ describes the orbital interactions and accounts for charge transfer and intramolecular polarization; iii) Pauli repulsion Δ*E*_Pauli_ corresponds to repulsive interactions between closed‐shell orbitals. In case of M06‐2X, this term also includes dispersion‐like attractive interactions, implicitly modelled in this functional. Δ*E*_Pauli_ can therefore be understood as responsible for steric repulsion.^[16]^

## Results and Discussion

### Reactivity of disubstituted tetrazines with ethylene

Tetrazines commonly applied in bioorthogonal click reactions can be classified in five different groups depending on the substitution pattern: alkyl‐alkyl, aryl‐aryl, aryl‐alkyl, mono‐aryl, or mono‐alkyl. In this study we focused on phenyl‐ and methyl‐substituted tetrazines as representative examples to get mechanistic insight regarding the various compound classes (Figure 1b).

Starting with the symmetric compounds 3,6‐diphenyl‐1,2,4,5‐tetrazine (**Ph_2_Tz**), 3,6‐dimethyl‐1,2,4,5‐tetrazine (**Me_2_Tz**), and non‐substituted 1,2,4,5‐tetrazine (**Tz**), we first studied the intrinsic reactivity in the gas phase with ethylene as a model dienophile with low reactivity, as frequently used in theoretical investigations of Diels–Alder cycloadditions.^[17]^

**Figure 2:**
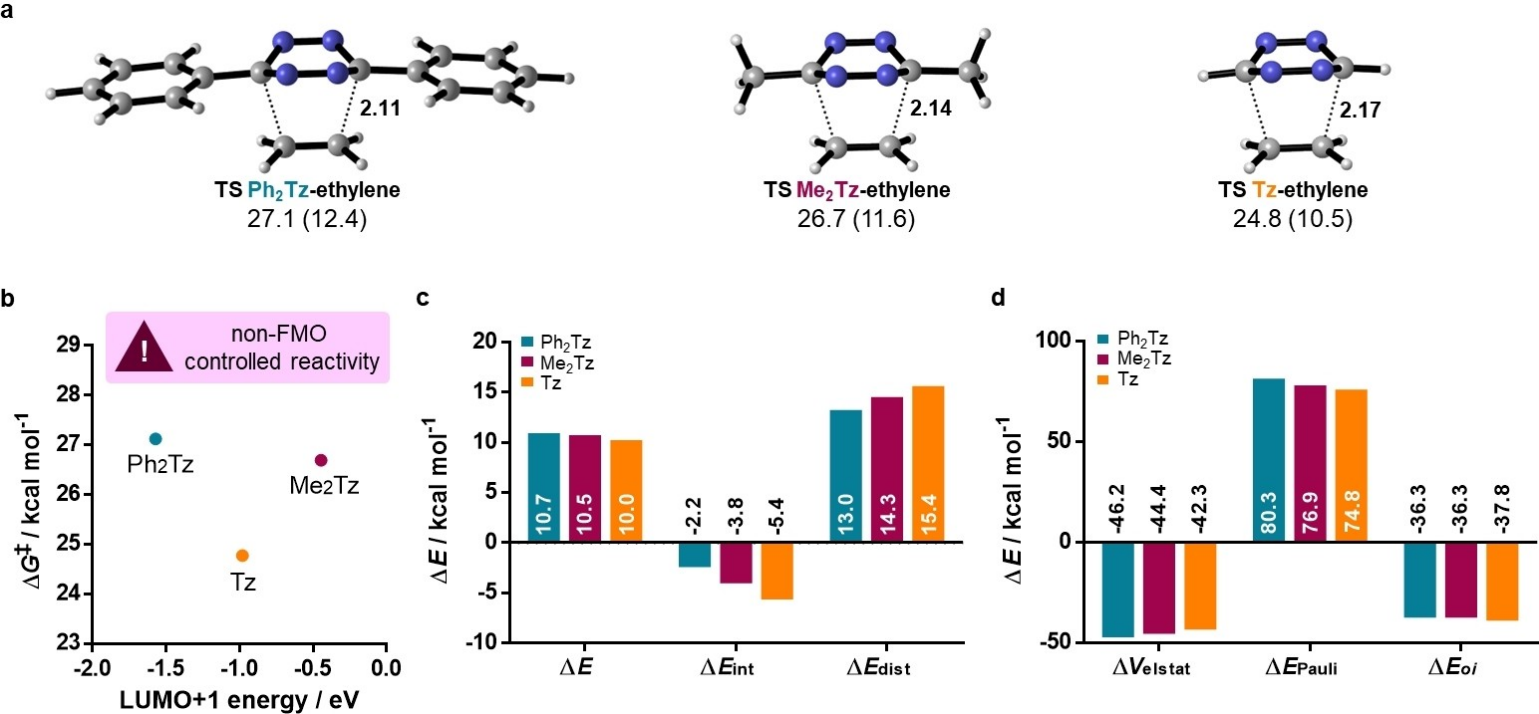
a) Transition state geometries and activation energies for the reaction of ethylene with **Ph_2_Tz**, **Me_2_Tz**, and **Tz**; distances are given in Å. Gibbs free energies of activation and electronic energies of activation (in parenthesis) are shown in kcal mol^−1^. b) Gibbs free energies of activation against LUMO+1 energies. c) Distortion/interaction and d) energy decomposition analyses at a consistent geometry with formation of C−C bond lengths of 2.25 Å.

Gibbs free energy barriers for the reaction with ethylene were calculated to be 27.1, 26.7, and 24.8 kcal mol^−1^ for **Ph_2_Tz**, **Me_2_Tz**, and **Tz**, respectively (Figure 2a). Thus, **Me_2_Tz** showed an unexpected higher reactivity than **Ph_2_Tz**. It contradicts reactivity predictions using FMO theory (Figure 2b) and experimental observations with other alkenes, with the diphenyl derivative being more reactive than dialkyl compounds.^[3]^

This unusual reactivity seems to be unique to ethylene, as introducing more steric demand on the alkene, for example, in *trans*‐but‐2‐ene, leads to a higher reactivity for **Ph_2_Tz** compared to **Me_2_Tz** (Figure S1 in the Supporting Information). To investigate this unexpected behavior, we conducted distortion/interaction (Figure 2c) and energy decomposition analyses (Figure 2d). These were performed at a consistent geometry for all three tetrazines, to exclude effects arising from differences in forming bond lengths at the respective transition states.[^14a^, ^18^] Thus, the bond length was frozen to 2.25 Å, at which electronic energies follow the same trend as the transition states. Distortion energies are the highest for unsubstituted tetrazine **Tz**, while **Ph_2_Tz** shows considerably lower distortion energies than both **Me_2_Tz** and **Tz**. The interaction energy of **Me_2_Tz** with ethylene is 1.6 kcal mol^−1^ more favorable than for **Ph_2_Tz**. The increased reactivity of **Me_2_Tz** compared to **Ph_2_Tz** was identified to result from a 3.4 kcal mol^−1^ higher Pauli repulsion in case of the diphenyl derivative. **Tz** was found to be the most reactive tetrazine due to the lowest Pauli repulsion. Δ*V*_elstat_ is more favorable for **Ph_2_Tz** than for **Me_2_Tz**, a supplemental qualitative view on this difference is presented in the Supporting Information (page S5).

Despite being heavily used to study Diels–Alder cycloadditions, ethylene is thus not suited as a reliable model dienophile to investigate the reactivity of tetrazines and should only be used if it is the dienophile of interest, which is not the case in any bioorthogonal application.

### Reactivity of disubstituted tetrazines with trans‐cyclooctene

Therefore, we turned to *trans*‐cyclooctene (**TCO**), the most commonly applied dienophile for bioorthogonal tetrazine ligations, due to the exceptionally high reaction rates that can be reached; a key criterion for *in vivo* applications in which low concentrations are encountered.

**Figure 3:**
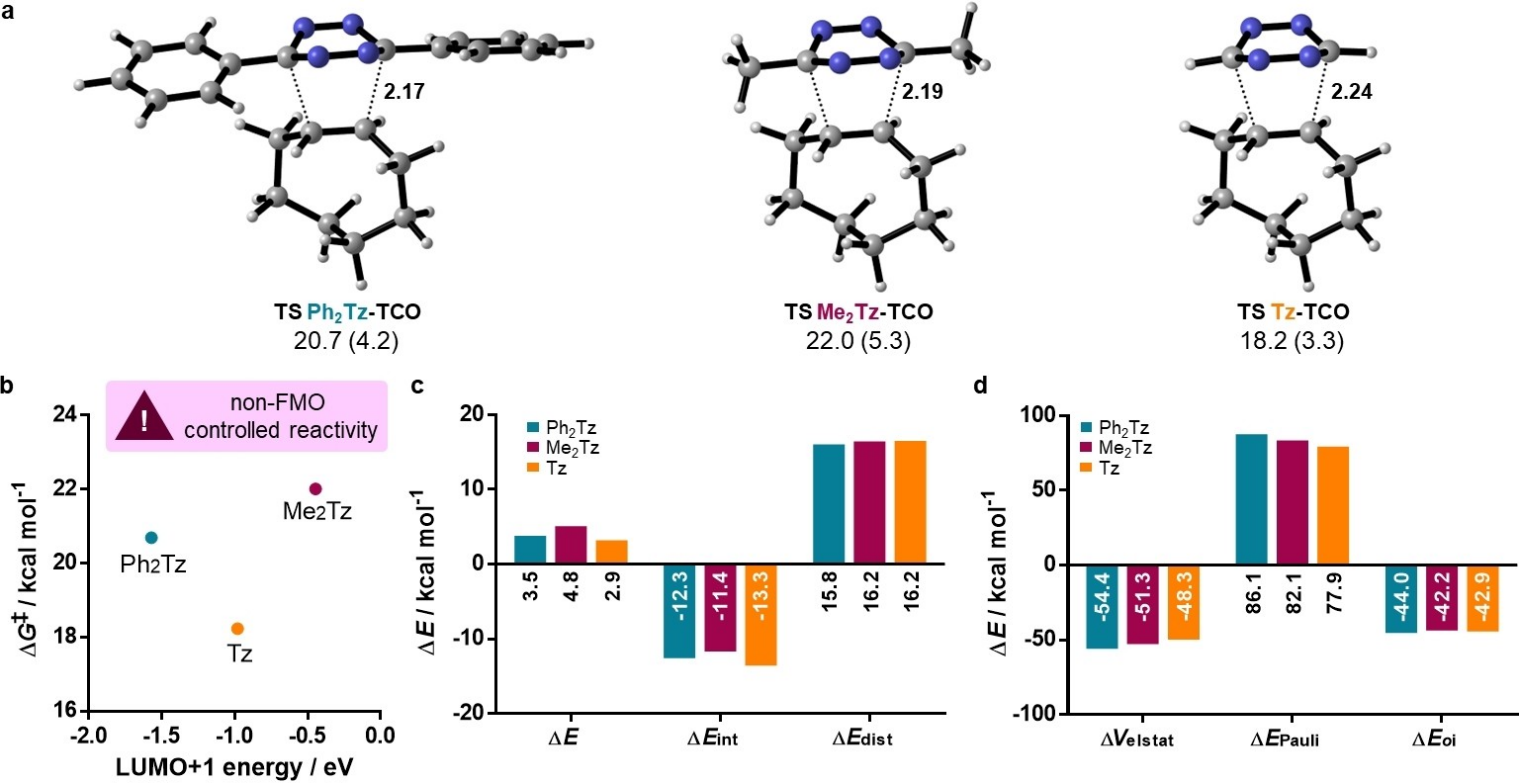
a) Transition state geometries and activation energies for the reaction of **TCO** with **Ph_2_Tz**, **Me_2_Tz**, and **Tz**; distances are given in Å. Gibbs free energies of activation and electronic energies of activation (in parenthesis) are shown in kcal mol^−1^. b) Gibbs free energies of activation against LUMO+1 energies. c) Distortion/interaction and d) energy decomposition analyses at a consistent geometry with formation of C−C bond lengths of 2.25 Å.

Gibbs free energies of activation were calculated to be 20.7, 22.0, and 18.2 kcal mol^−1^ for **Ph_2_Tz**, **Me_2_Tz**, and **Tz**, respectively, following experimentally observed trends (Figure 3a). Similar to the reaction with ethylene, reactivity was found to be not controlled by FMO interactions (Figure 3b). Next, distortion/interaction analyses and EDA were performed at a consistent geometry of a forming bond length of 2.25 Å (Figure 3c and d). **Ph_2_Tz** showed a slightly lower distortion energy compared to **Me_2_Tz** and **Tz** showed the highest. This can be attributed to the stabilizing interactions of the substituents on the tetrazine carbons that get distorted towards a tetrahedral geometry during the reaction. The phenyl substituent can stabilize the partially positive carbon center through mesomeric effects, leading to a lower distortion energy. Alkyl substituents stabilize through weaker hyperconjugation, while in case of **Tz** no stabilization is possible.^[19]^ Interaction energies are strongest for **Tz**, caused by a considerably lowered Pauli repulsion which results in the highest reactivity. As observed for the reaction with ethylene, **Ph_2_Tz** was calculated to show a higher Pauli repulsion than **Me_2_Tz**. However, this is compensated by more favorable electrostatic and orbital interactions causing **Me_2_Tz** to exhibit the lowest reactivity. Based on this data, the steric demand of tetrazine substituents (i. e., Pauli repulsion at a given distance) follows the order of aryl > primary alkyl > hydrogen.

### Reactivity of mono‐substituted tetrazines

**Figure 4:**
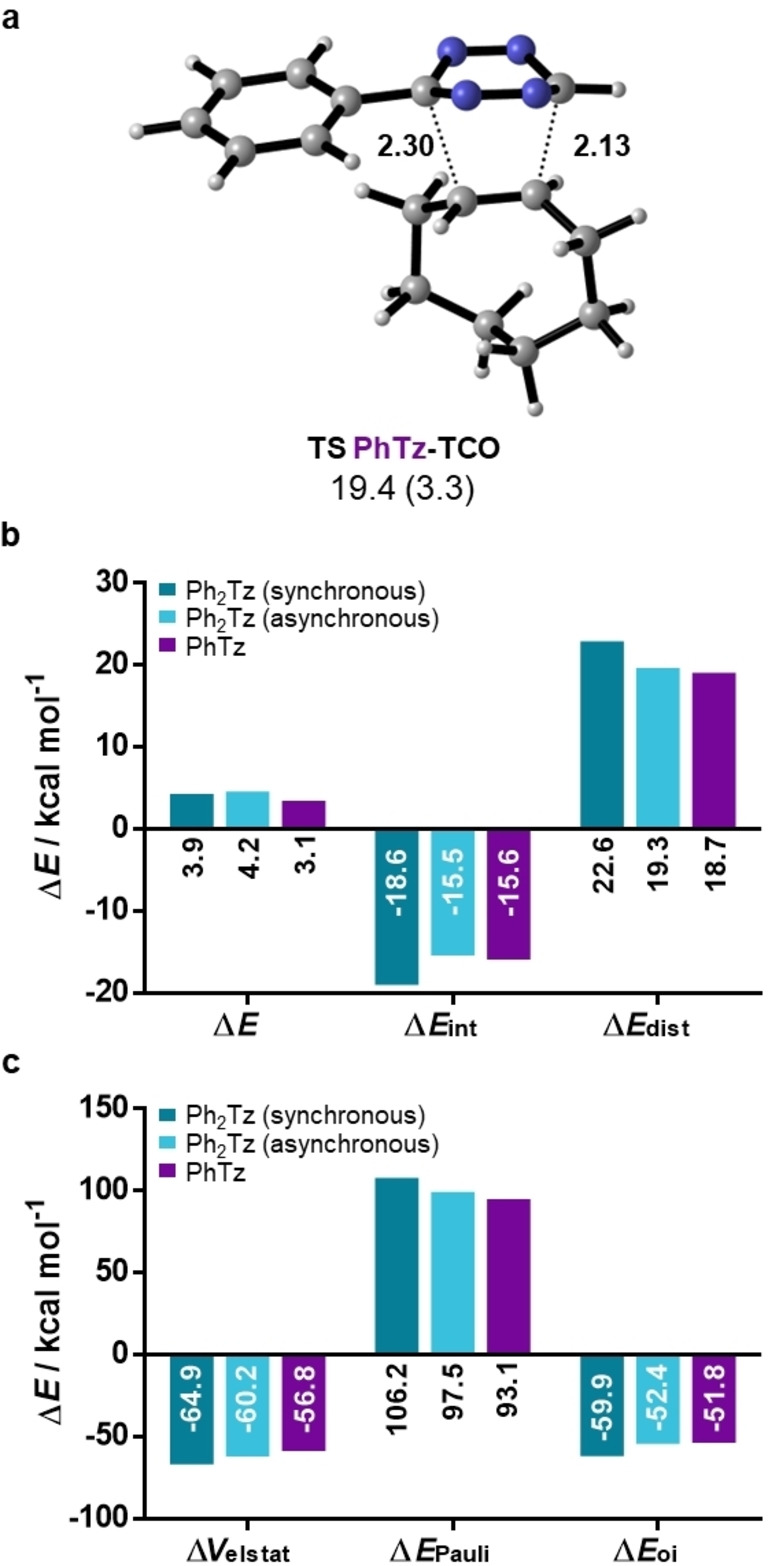
a) Transition state geometries and activation energies for the reaction of **TCO** with **PhTz**; distances are given in Å. Gibbs free energies of activation and electronic energies of activation (in parenthesis) are shown in kcal mol^−1^; b) Distortion/interaction and **c)** energy decomposition analyses.

Having investigated symmetrical cases, we then turned to non‐symmetric scaffolds. In particular, mono‐aryl substituted tetrazines are commonly used in bioorthogonal chemistry due to their high reactivity.^[4]^ Therefore, we compared 3‐phenyl‐1,2,4,5‐tetrazine (**PhTz**) to the symmetric **Ph_2_Tz**. Due to the different substituents, the transition state geometry of **PhTz** with **TCO** shows a high degree of asynchronicity with forming bond lengths differing by 0.17 Å (Figure 4a), while the reaction of **Ph_2_Tz** is completely synchronous (Figure 3a). Free energy barriers were calculated to be 19.4 and 20.7 kcal mol^−1^ for **PhTz** and **Ph_2_Tz**, respectively, which is in agreement with experimentally observed reactivity trends.^[20]^ Again, the reactivity was found to be non‐FMO controlled, with the more reactive **PhTz** having a higher LUMO+1 energy (−1.38 eV) than Ph_2_Tz (−1.57 eV). Distortion/interaction analysis and EDA revealed that at the transition state (TS) geometry, **PhTz** has a considerably lowered distortion energy compared to Ph_2_Tz, overall resulting in a lower barrier (Figure 4b). The interaction energy is more favorable in the case of **Ph_2_Tz**, rooted in a much stronger orbital and electrostatic interaction (Figure 4c). In contrast, Pauli repulsion is lower for **PhTz**. However, comparing transition states with vastly different asynchronicity to each other can produce misleading interpretations. For example, in Diels–Alder reactions a more asynchronous approach can lead to both reduced orbital interactions and reduced Pauli repulsion.^[21]^ To understand the difference between **PhTz** and **Ph_2_Tz** in more detail, we performed an additional analysis. For **Ph_2_Tz**, a theoretical TS‐like structure, mimicking the asynchronous reaction of **PhTz** with forming bond distances of 2.13 and 2.30 Å, was created. This was achieved by optimizing the structure with forming bond lengths fixed to these values. This allows us to understand the origin of (a)synchronicity and the driving forces for the lowered barrier as observed for **PhTz**.

Tilting **Ph_2_Tz** from the synchronous to the asynchronous approach results in a slightly higher barrier of 4.2 kcal mol^−1^ compared to 3.9 kcal mol^−1^ in the natural TS. The distortion energy is reduced, albeit still being higher than for **PhTz**. The interaction energy is also lowered and slightly less favorable than for **PhTz**, which is caused by a higher Pauli repulsion. An additional analysis based on a **PhTz**‐**TCO** transition state fixed to the synchronous values of **Ph_2_Tz** provided the same qualitative results (see the Supporting Information). In essence, hydrogen as the smallest possible substituent leads to a reduced Pauli repulsion, as already demonstrated for the symmetrically substituted tetrazines. This allows for an asynchronous transition state geometry where the distortion energy is significantly reduced compared to tetrazines with more bulky phenyl substituents. This is in agreement with the “origin of asynchronicity in Diels–Alder reactions” study by Vermeeren, Hamlin, and Bickelhaupt, in which the same mechanism was described for rate acceleration in Lewis‐acid‐catalyzed Diels–Alder reactions.^[21]^

## Conclusion

In summary, we have shown that, despite being commonly used, ethylene is not a reliable model dienophile, potentially leading to results that cannot be used to predict reactions with other alkenes. We thus strongly suggest using ethylene only in computational studies if the reactivity of this particular molecule is of interest.

Moreover, we show that the reactivity of tetrazines in Diels–Alder cycloadditions is often not controlled by FMO interactions. In fact, we have revealed that even the higher reactivity of 3,6‐diphenyl‐1,2,4,5‐tetrazine compared to 3,6‐dimethyl‐1,2,4,5‐tetrazine is rooted primarily in a lower distortion energy and an increased electrostatic attraction, whereas improved orbital interaction plays a negligible role.

The high reactivity of unsubstituted 1,2,4,5‐tetrazine is caused by reduced Pauli repulsion, which manifests itself in monosubstituted 1,2,4,5‐tetrazines in a highly asynchronous transition state geometry. While sacrificing orbital interaction, this causes a reduced distortion energy, resulting in a lowered activation energy and high reactivity.

## Conflict of interest

The authors declare no conflict of interest.

## Supporting information

As a service to our authors and readers, this journal provides supporting information supplied by the authors. Such materials are peer reviewed and may be re‐organized for online delivery, but are not copy‐edited or typeset. Technical support issues arising from supporting information (other than missing files) should be addressed to the authors.

Supporting Information

## Data Availability

The data that support the findings of this study are available in the supplementary material of this article.
