# Supplementary material for: Substituent Effects in Bioorthogonal Diels–Alder Reactions of 1,2,4,5‐Tetrazines
Source: Chemistry. 2023 Apr 13;29(29):e202300345. doi: 10.1002/chem.202300345 (PMC10946812; doi:10.1002/chem.202300345)
Supplement: Supplementary file 1 — Supporting Information [file CHEM-29-0-s001.pdf]

Chemistry–A European Journal

Supporting Information

Substituent Effects in Bioorthogonal Diels–Alder Reactions of 1,2,4,5-Tetrazines

Nicole Houszka, Hannes Mikula, and Dennis Svatunek*

Table of contents

1) Computational methods and results S2

2) Reaction with *trans*-2-butene S3

3) EDA results S4

4) Electrostatic potential analysis S5

5) References S6

1) Computational methods and results

Theoretical calculations were performed using the software package Gaussian16 Rev. A.03.[1] Geometry optimizations and frequency analyses were performed using the M06-2X density functional and def2-TZVP basis set. A quasi-harmonic correction to entropy was applied by setting all frequencies below 100 cm^{-1} to 100 cm^{-1} using GoodVibes.[2] Single imaginary frequencies corresponding to the desired reaction coordinates were obtained only in the case of transition state (TS) calculations. No imaginary frequencies were obtained for all other structures. Distortion/interaction analysis was performed in ADF as part of the energy decomposition analysis and matched those obtained in Gaussian calculated using autoDIAS.[3] Energy decomposition analysis (EDA) was performed using PyFrag 2019.[4]

"Consistent geometry" structures with were achieved by performing an optimization with frozen C–C forming bond lengths using the `opt=modredundant` keyword in Gaussian 16.

The calculated energies are summarized in Table S1. Coordinates of all optimized geometries are provided as *.xyz files.

Table S1. M06-2X/def2-TZVP calculated energies for all investigated Tz and the transition states for the reactions with **TCO** and **ethylene**.

Structure	ΔE (hartree)	ZPE (hartree)	ΔH_{298} (hartree)	ΔG_{298} (hartree)
**TCO**	-313.202772	0.205232	-312.989107	-313.028661
**ethylene**	-78.571977	0.051412	-78.516586	-78.542078
**Ph_2Tz**	-758.416193	0.215214	-758.186792	-758.240037
**Me_2Tz**	-374.951483	0.107156	-374.835678	-374.876217
**Tz**	-296.317274	0.052381	-296.259833	-296.292235
**PhTz**	-527.366844	0.133898	-527.223422	-527.266864
**TS-Ph_2Tz-ethylene**	-836.968340	0.270150	-836.682077	-836.738893
**TS-Me_2Tz-ethylene**	-453.504987	0.162720	-453.332097	-453.375758
**TS-Tz-ethylene**	-374.872533	0.107593	-374.757979	-374.794834
**TS-Ph_2Tz-TCO**	-1071.612277	0.422702	-1071.167787	-1071.235723
**TS-Me_2Tz-TCO**	-688.145780	0.315624	-687.814508	-687.869806
**TS-Tz-TCO**	-609.514871	0.259944	-609.242292	-609.291845
**TS-PhTz-TCO**	-840.564301	0.341334	-840.205795	-840.264551

2) Reaction with *trans*-2-butene

Figure S1 shows transition state geometries and energies of activation for the reaction between *trans*-2-butene and **Ph_2Tz** or **Me_2Tz**. In contrast to the reaction with ethylene, **Me_2Tz** shows a lower reactivity than **Ph_2Tz**. Table S2 lists the calculated energies for the involved species.

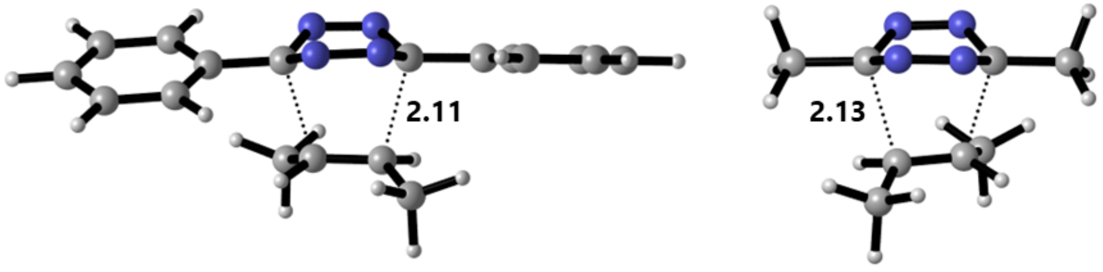

Figure S1. Transition state geometries for the reactions between **Ph_2Tz** and **Me_2Tz** with *trans*-2-butene. Gibbs free energy of activation and electronic energy of activation (in parenthesis) are given below in kcal/mol.

Table S2. M06-2X/def2-TZVP calculated energies for the transition states for the reactions with *trans*-2-butene.

Structure	ΔE (hartree)	ZPE (hartree)	ΔH_{298} (hartree)	ΔG_{298} (hartree)
***trans*-2-butene**	-157.195008	0.108561	-157.080038	-157.113827
**TS-Me_2Tz-butene**	-532.122429	0.219362	-531.890026	-531.939651
**TS-Ph_2Tz-butene**	-915.588872	0.326908	-915.243147	-915.305263

3) EDA results

EDA were performed in ADF using structures obtained in Gaussian at the M06-2X/def2-TZVP level of theory. Table 3 lists the result of EDA at a consistent geometry with forming bond lengths frozen to 2.25 Å.

Table S3. Results of EDA at consistent geometry of 2.25 Å forming bond lengths at the M06-2X/TZ2P//M06-2X/def2-TZVP level of theory.

Structure	ΔE (kcal/mol)	ΔE_{int} (kcal/mol)	ΔV_{elstat} (kcal/mol)	ΔE_{Pauli} (kcal/mol)	ΔE_{oi} (kcal/mol)	ΔE_{dist} (kcal/mol)
**TS-Ph_2Tz-ethylene***	10.7	-2.2	-46.2	80.3	-36.3	13.0
**TS-Me_2Tz-ethylene***	10.5	-3.8	-44.4	76.9	-36.3	14.3
**TS-Tz-ethylene***	10.0	-5.4	-42.3	74.8	-37.8	15.4
**TS-Ph_2Tz-TCO***	3.477	-12.282	-54.354	86.068	-43.997	15.759
**TS-Me_2Tz-TCO***	4.775	-11.382	-51.253	82.102	-42.231	16.157
**TS-Tz-TCO***	2.932	-13.29	-48.263	77.894	-42.921	16.222
**TS-PhTz-TCO***	1.753	-5.629	-35.66	54.102	-24.071	7.382

* Artificial transition state like geometry with forming bond lengths frozen to 2.25 Å.

For **PhTz** and **Ph_2Tz** additional EDA results were obtained by performing the analysis either at the bond lengths of the synchronous **Ph_2Tz** or asynchronous **PhTz** transition state. **TS-Ph_2Tz-TCO** is the transition state geometry of **Ph_2Tz** with **TCO** which is synchronous with a forming bond length of 2.17 Å. **TS-PhTz-TCO synchronous** is an artificial transition state like geometry where the forming bond lengths are fixed to 2.17 Å. **TS-Ph_2Tz-TCO** is the transition state geometry of **PhTz** with **TCO** which is asynchronous with forming bond lengths of 2.13 and 2.30 Å. **TS-Ph_2Tz-TCO asynchronous** is an artificial transition state-like geometry where the forming bond lengths are fixed to 2.13 and 2.30 Å.

Table S4. Results of EDA at transition state geometry and alternative geometry for **PhTz** and **Ph_2Tz** at the M06-2X/TZ2P//M06-2X/def2-TZVP level of theory.

Structure	ΔE (kcal/mol)	ΔE_{int} (kcal/mol)	ΔV_{elstat} (kcal/mol)	ΔE_{Pauli} (kcal/mol)	ΔE_{oi} (kcal/mol)	ΔE_{dist} (kcal/mol)
**TS-Ph_2Tz-TCO**	3.924	-18.632	-64.925	106.176	-59.883	22.556
**TS-Ph_2Tz-TCO asynchronous**	4.24	-15.05	-60.15	97.49	-52.4	19.29
**TS-PhTz-TCO**	3.096	-15.582	-56.83	93.068	-51.82	18.678
**TS-PhTz-TCO synchronous**	3.39	-19.25	-61.73	101.92	-59.44	22.64

In the synchronous transition states **TS-Ph_2Tz-TCO** and **TS-PhTz-TCO synchronous**, **PhTz** is favored due to a stronger interaction energy, once again caused by a significantly lower Pauli repulsion. This is in agreement with the analysis at the respective transition state geometry and at the asynchronous transition states.

4) Electrostatic potential analysis

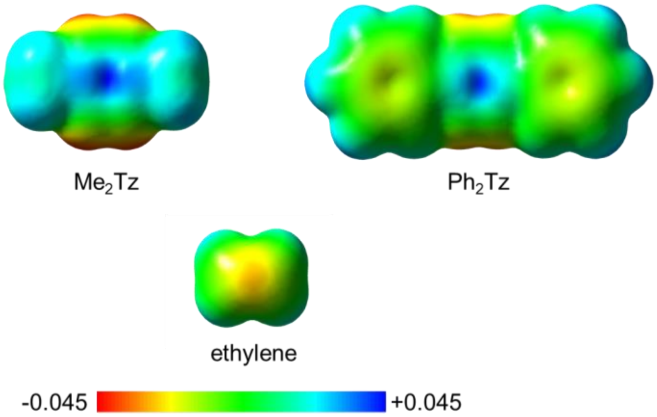

Figure S2. Electrostatic potential projected on a total electron density surface (isovalue: 0.001) for Me2Tz, Ph2Tz, and ethylene at the consistent TS geometry with a forming bond length of 2.25 Å. Views are along the reaction axis.

EDA (page S4) revealed a 1.8 kcal/mol more favorable electrostatic interaction of **Ph_2Tz** with ethylene compared to **Me_2Tz** at a consistent TS geometry with a forming bond length of 2.25 Å. To shine light on this difference, a qualitative analysis of the electrostatic potential (ESP) was conducted (Figure S2). Ethylene shows a slightly negative potential in the middle of the double bond, with slightly positive potential next to it and more positive potential on the sides, caused by the polarized CH bonds. Both tetrazines show a strong positive potential in the middle of the tetrazine ring, which matches up with the negative potential of ethylene. Phenyl rings exhibit a negative ESP which can interact with the slight positive potential on the side of ethylene, thus causing an increased electrostatic attraction.

5) References

1. Frisch, M.; Trucks, G.; Schlegel, H.; Scuseria, G.; Robb, M.; Cheeseman, J.; Scalmani, G.; Barone, V.; Mennucci, B.; Petersson, G., Gaussian 16 Revision A. 03, 2016. *Gaussian Inc. Wallingford CT* **2009**.
2. Luchini, G.; Alegre-Requena, J. V.; Funes-Ardoiz, I.; Paton, R. S., GoodVibes: automated thermochemistry for heterogeneous computational chemistry data. *F1000Research* **2020,** *9*.
3. Svatunek, D.; Houk, K. N., autoDIAS: a python tool for an automated distortion/interaction activation strain analysis. *J Comput Chem* **2019,** *40* (28), 2509-2515.
4. Sun, X.; Soini, T. M.; Poater, J.; Hamlin, T. A.; Bickelhaupt, F. M., PyFrag 2019-Automating the exploration and analysis of reaction mechanisms. *J Comput Chem* **2019,** *40* (25), 2227-2233.
